# Placental epigenetic clocks: estimating gestational age using placental DNA methylation levels

**DOI:** 10.18632/aging.102049

**Published:** 2019-06-24

**Authors:** Yunsung Lee, Sanaa Choufani, Rosanna Weksberg, Samantha L. Wilson, Victor Yuan, Amber Burt, Carmen Marsit, Ake T. Lu, Beate Ritz, Jon Bohlin, Håkon K. Gjessing, Jennifer R. Harris, Per Magnus, Alexandra M. Binder, Wendy P. Robinson, Astanand Jugessur, Steve Horvath

**Affiliations:** 1 Department of Genetics and Bioinformatics, Norwegian Institute of Public Health, Oslo, Norway; 2 Genetics and Genome Biology Program, Research Institute, The Hospital for Sick Children, Toronto, Ontario, Canada; 3 Genetics and Genome Biology Program, Research Institute, The Hospital for Sick Children and Institute of Medical Science, University of Toronto, Toronto, Ontario, Canada; 4 Department of Medical Genetics, University of British Columbia, Vancouver, British Columbia, Canada; 5 B.C. Children’s Hospital Research Institute, Vancouver, British Columbia, Canada; 6 Department of Environmental Health, Rollins School of Public Health, Emory University, Atlanta, GA 30322, USA; 7 Department of Human Genetics, David Geffen School of Medicine, University of California Los Angeles, Los Angeles, CA 90095, USA; 8 Department of Epidemiology, University of California Los Angeles, Los Angeles, CA 90095, USA; 9 Centre for Fertility and Health, Norwegian Institute of Public Health, Oslo, Norway; 10 Department of Global Public Health and Primary Care, University of Bergen, Bergen, Norway; 11 Department of Biostatistics, Fielding School of Public Health, University of California Los Angeles, Los Angeles, CA 90095, USA

**Keywords:** DNA methylation, epigenetic clock, placenta, gestational age

## Abstract

The human pan-tissue epigenetic clock is widely used for estimating age across the entire lifespan, but it does not lend itself well to estimating gestational age (GA) based on placental DNAm methylation (DNAm) data. We replicate previous findings demonstrating a strong correlation between GA and genome-wide DNAm changes. Using substantially more DNAm arrays (n=1,102 in the training set) than a previous study, we present three new placental epigenetic clocks: 1) a robust placental clock (RPC) which is unaffected by common pregnancy complications (e.g., gestational diabetes, preeclampsia), and 2) a control placental clock (CPC) constructed using placental samples from pregnancies without known placental pathology, and 3) a refined RPC for uncomplicated term pregnancies. These placental clocks are highly accurate estimators of GA based on placental tissue; e.g., predicted GA based on RPC is highly correlated with actual GA (r>0.95 in test data, median error less than one week). We show that epigenetic clocks derived from cord blood or other tissues do not accurately estimate GA in placental samples. While fundamentally different from Horvath’s pan-tissue epigenetic clock, placental clocks closely track fetal age during development and may have interesting applications.

## Introduction

Gestational age (GA) of the fetus is used to forecast the date of delivery, optimize prenatal care, and monitor the growth and development of the fetus relative to other pregnancies. Short GA at delivery impacts neonatal morbidity and mortality [1–3], as well as brain development [4–6]. Thus, accurate classification of the fetus may help predict neonatal risk. In this regard, the World Health Organization defined extremely preterm (<28 weeks of gestation), very preterm (28-32 weeks of gestation) and moderate or late preterm (32-37 weeks of gestation) birth to reflect the newborn’s developmental stage [7].

Traditional methods for estimating GA include early obstetric ultrasound measures or calculations based on the last menstrual period (LMP) [8]. The early ultrasound method estimates GA based on the visible fetal size (e.g., crown-rump length during the first trimester [9–11] or biparietal diameter after the second trimester [12–15]). The LMP method calculates GA based on the time elapsed since the known first day of the LMP. The early ultrasound method is widely accepted as the gold standard due to its higher accuracy [16] but is not routinely available in low and middle-income countries. More accurate classification of GA at birth may help predict neonatal risk for adverse outcomes and measure GA more accurately than through the assessment of physical and neurological features of the newborn, especially when early ultrasound measures are lacking, or the infant is growth-restricted but not preterm.

Here, we aim to develop a new molecular estimator of GA based on placental tissue samples that is more accurate than the previous clock [17]. Earlier studies have revealed profound molecular changes in placental chorionic villi, the placental structures that project into maternal decidua and are bathed in maternal blood, during gestation [18–22]. We focus on placental DNA methylation (DNAm) data, because prior work demonstrated that accurate estimators of chronological age (epigenetic clocks) can be developed based on DNAm levels from a variety of tissues [23], that one can estimate GA based on DNAm data derived from umbilical cord blood samples [24,25], and most pertinently that one can estimate GA based on placental methylation data (Mayne et al. 2017) [17]. Our study provides more accurate placenta-based GA estimators (i.e. placental epigenetic clocks) than those developed previously, because we use a substantially larger sample for our training set (more than six times larger than that of Mayne et al. 2017). We aim to develop three different placental epigenetic clocks: 1) a “robust placental clock” (RPC) that is largely unaffected by pregnancy conditions (e.g., preeclampsia, gestational diabetes, and trisomy), 2) a “control placental clock” (CPC), tailor-made for measuring GA in normal pregnancies, and 3) a “refined RPC”, trained for uncomplicated term (GA>36) pregnancies. For the RPC, we purposely included placental samples from a variety of pregnancy complications in the training data (e.g., hypertension or diabetes) as well as congenital abnormalities (e.g., trisomy 13, 18 and 21).

## RESULTS

### Placental DNA methylation data

We downloaded publicly available DNAm data from Gene Expression Omnibus (GEO, https://www.ncbi.nlm.nih.gov/geo/; Table 1) that assessed DNAm levels in placental tissues. Eighteen datasets used the Illumina HumanMethylation 450K BeadChip (450K) platform and one used the more recent Illumina MethylationEPIC BeadChip (EPIC) array. Our analyses focused on the 441,870 autosomal CpG probes that are shared between the two Illumina platforms such that the resulting GA estimators (RPC and CPC) would be applicable to data from both platforms.

**Table 1:** Description of the publicly available placental DNAm data.

GEO Number	Placental tissue type	GEO submitter	N	Platform		Normalization method	Probe exclusion criteria^11^	GA range (weeks)
**Training data**								
GSE71678	Fetal side, near the cord insertion	Marsit et al.	343	450K^2^		funNorm^4^	SC, CH, SNP, DP	30-42
GSE75248	Fetal side	Marsit et al.	334	450K^2^		funNorm^4^	SC, CH, SNP, DP	37-42
GSE71719	Fetal side, near the cord insertion	Marsit et al.	44	450K^2^		noob^5^	SC, CH, SNP, DP	37-41
RL^1^	Fetal side, chorionic villi	-	121	450K^2^		funNorm^4^	SC	14-42
GSE100197	Fetal side, chorionic villi	Robinson et al.	16	450K^2^		SWAN^6^	SC, SNP, DP, MB	26-39
GSE108567	Fetal side, chorionic villi	Robinson et al.	7	450K^2^		SWAN^6^	SC, CH, SNP, DP, BR	29-38
GSE69502	Fetal side, chorionic villi	Robinson et al.	7	450K^2^		SWAN^6^	SC, CH, SNP, DP, BR	16-24
GSE74738	Fetal side, chorionic villi	Robinson et al.	8	450K^2^		SWAN^6^	SC, CH, SNP, DP, BR	6-13
GSE115508	Fetal side, chorionic villi	Robinson et al.	44	EPIC^3^		funNorm^4^	SC, CH, SNP, DP, BR	28-37
GSE44667	Fetal side, chorionic villi	Robinson et al.	27	450K^2^		SWAN^6^	SC, SNP, DP, MB	25-37
GSE49343	Fetal side, chorionic villi	Robinson et al.	13	450K^2^		SWAN^6^	SC, SNP, DP	5-39
GSE42409	Fetal side, chorionic villi	Robinson et al.	4	450K^2^		SWAN^6^	SC, SNP, DP	26-33
GSE120250	Fetal side, near the cord insertion	Weksberg et al.	86	450K^2^		GenomeStudioNorm^7^	SC, SNP, DP	35-41
GSE98224	Fetal side	Cox et al.	48	450K^2^	SWAN^6^		SC, SNP, DP, MB	27-41
**Test data**								
GSE70453	Maternal side, decidua near the cord	Binder et al.	82	450K^2^		BMIQ^8^	SC, CR, SNP	35-42
GSE73375	Fetal side	Fry et al.	36	450K^2^		quanNorm^9^	DP	22-41
GSE75196	Fetal side	Chiu et al.	24	450K^2^		dasen^10^	SC, SNP, DP, BR	32-40
GSE76641	Fetal side, chorionic villi	Slieker et al.	4	450K^2^		funNorm^4^	SC, SNP, DP, BR	9-22
GSE66210	Fetal side, chorionic villi	Bojesen et al.	41	450K^2^		GenomeStudioNorm^7^	-	11-15

^1^ Placental DNAm data generated from the Robinson laboratory at the University of British Columbia (Vancouver, BC, Canada); The data for which is publicly available as part of the GEO data sets listed below.

^2^ 450K: Illumina Infinium HumanMethylation450 BeadChip

^3^ EPIC: Infinium MethylationEPIC BeadChip

^4^ funNorm: Functional normalization [27]

^5^ noob: Normal-exponential out-of-band [29]

^6^ SWAN: Subset-quantile within array normalization [28]

^7^ GenomeStudioNorm: Genome Studio normalization

(details available in the GenomeStudio Methylation Module v1.8 User Guide, https://www.illumina.com/content/dam/illumina-support/documents/documentation/software_documentation/genomestudio/genomestudio-2011-1/genomestudio-methylation-v1-8-user-guide-11319130-b.pdf)

^8^ BMIQ: Beta-mixture quantile dilation [30]

^9^ quanNorm: Quantile normalization [31,32]

^10^ dasen: Data-driven separate normalization [33]

^11^ Probe exclusion criteria

SC: Sex chromosome, CH: Cross-hybridizing, SNP: Single nucleotide polymorphism, DP: Detection P-value < 0.01, MB: Missing beta > 5%, and BR: Bead replicates < 3.

### Robust placental clock (RPC)

**Figure 1:**
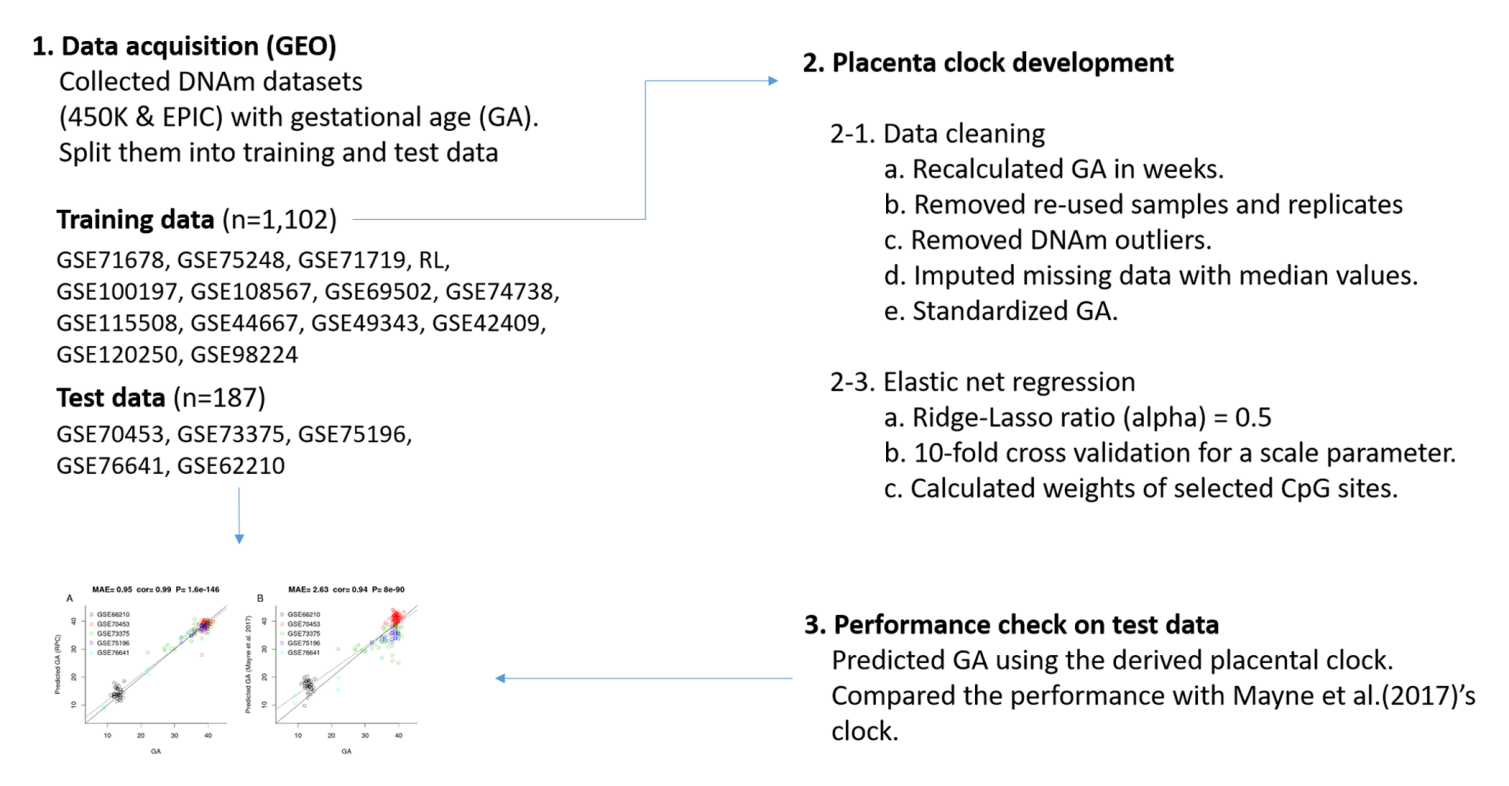
Flow chart of the RPC development.

An overview of our analysis is presented in Figure 1. We developed the RPC using several placental DNAm datasets (training n=1,102, Table 1, Figure 1). We regressed GA (dependent variable) on DNAm levels of CpG sites using a penalized regression model (elastic net regression [26]). The elastic net regression model automatically selected 558 CpG sites for the RPC model (Supplementary File 1). Predicted GA is a weighted average of DNAm levels at these 558 CpGs, where the weights are the regression coefficients.

**Figure 2:**
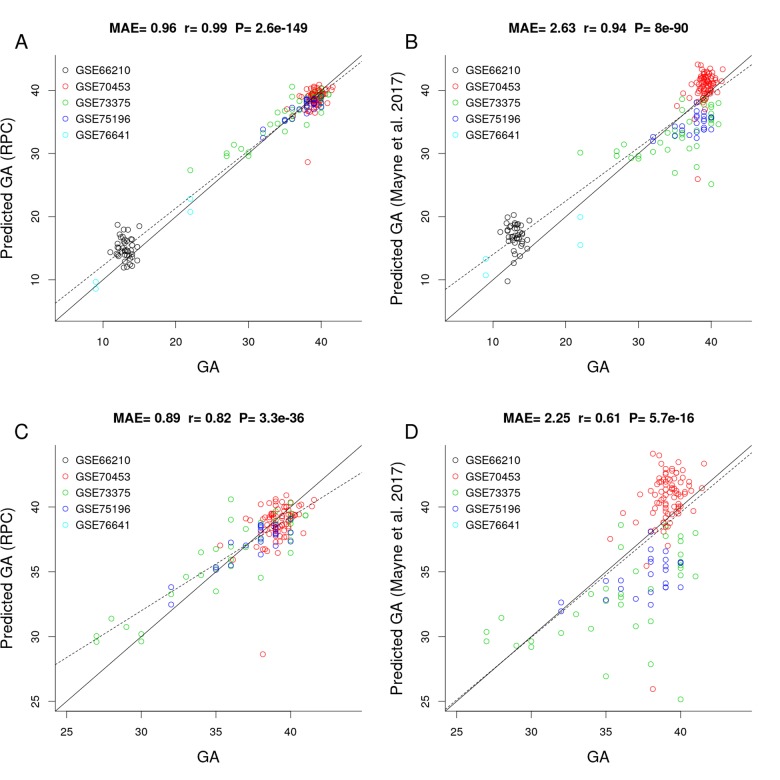
**Gestational age estimation of the RPC and Mayne et al. (2017)’s placental clock.** (**A**) Scatter plot between observed GA and DNAm-predicted GA (RPC) across all trimesters. (**B**) Scatter plot between observed GA and DNAm-predicted GA (Mayne et al. 2017) across all trimesters. (**C**) Zoom-in on panel **A** restricting GA > 25 weeks. (**D**) Zoom-in on panel B restricting GA > 25 weeks.

Figure 2 shows the results of a comparison between the RPC and the placental clock from Mayne et al. (2017) in independent test data (test n=187, Table 1). The predictive accuracy of the placental clocks was quantified using the median absolute error (MAE, defined as the median absolute deviation between predicted GA and observed GA), and the degree of the linear association between predicted GA and observed GA was measured using the Pearson correlation coefficient (r). According to both measures, the RPC (MAE=0.96 weeks; 95% confidence interval (CI) [0.88, 1.19], r=0.99; 95% CI [0.98, 0.99]) outperformed Mayne’s placental clock (MAE=2.63 weeks; 95% CI [2.17, 3.01], r=0.94; 95% CI [0.92, 0.96]). Note that Mayne’s placental clock underestimated GA in two data sets: GSE73375 (green dots) and GSE75196 (blue dots), and overestimated GA in two other data sets: GSE66210 (black) and GSE70453 (red).

The advantage of the RPC is particularly pronounced in later gestation, e.g., when restricting the analysis to placental samples with GA > 25 weeks, the RPC (MAE=0.89 [0.73, 1.02], r=0.82 [0.76, 0.87]) greatly outperforms Mayne's clock (MAE=2.25 [1.9, 2.63], r=0.61 [0.05,0.71], Figure 2C and 2D).

As expected by its construction, the RPC predicted GA accurately even in placental samples with adverse pregnancy conditions such as preeclampsia, gestational diabetes, and trisomy 13, 18 or 21 (Supplementary Figure S1). However, Mayne’s placental clock underestimated GA in placental samples from preeclampsia cases and overestimated GA in cases of gestational diabetes and trisomy (Supplementary Figure S1). In case of trisomy, the RPC (MAE=2.26 [1.63, 2.88], r=0.12 [-0.25, 0.46]) was more accurate than Mayne’s clock (MAE=3.99 [3.35, 5.4], r=0.02 [-0.34, 0.39]) but still showed a slight overestimation. The RPC's GA estimate was not associated with fetal sex (Supplementary Figure S2). We could not evaluate the effect of ethnicity because our test data did not include ethnic information except for GSE73375 (n=36, Supplementary Figure S3).

The training data used in the construction of the RPC employed several different normalization methods: functional normalization (funNorm [27],), subset-quantiles within arrays (SWAN [28],) and the normal-exponential out-of-band (noob [29],) approach. This lack of uniformity in normalization methods in the training data has a statistical advantage: it makes it more likely that the RPC will be robust with respect to different normalization methods. In support of this, we found that the RPC validated in test data that were normalized using various methods: beta-mixture quantile dilation (BMIQ [30],), quantile normalization (quanNorm [31,32],), data-driven separate normalization (dasen [33],) as detailed in Table 1.

### Control placental clock (CPC)

We trained the CPC on placental samples (training n=963, Table 1) that had been designated as "control" samples. Hence, placental samples with higher GA were probably from relatively normal pregnancies. However, placental samples with lower GA might contain samples that would be considered abnormal (i.e. premature rupture of membranes, spontaneous premature labor) but minimal placental pathology relative to preeclampsia cases. The analysis flow was identical as for the RPC, except for the composition of the training and test sets (Supplementary Figure S4). The elastic net regression model used for the CPC automatically selected 546 CpG sites (Supplementary File 1).

**Figure 3:**
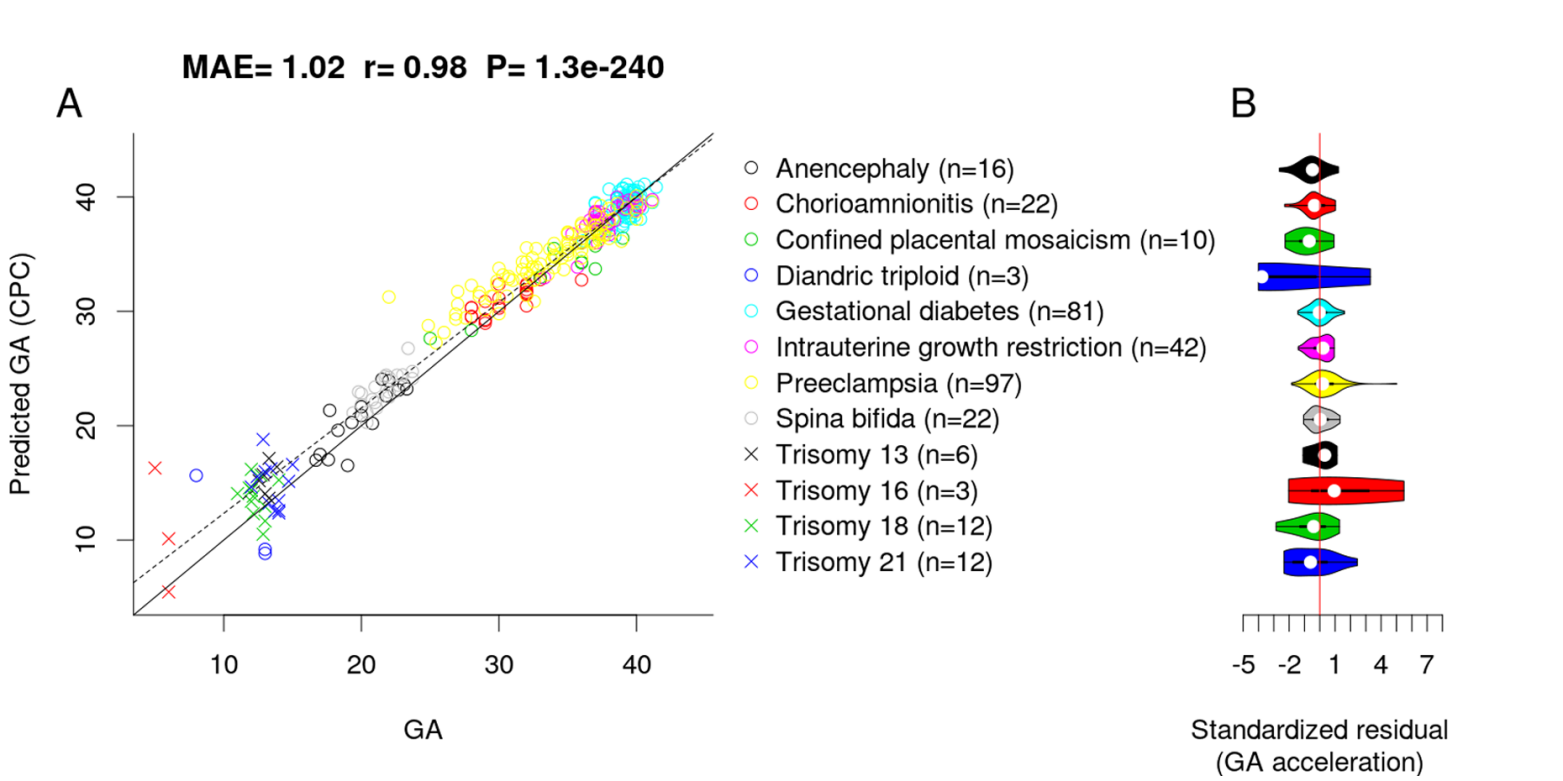
**Effect of pregnancy condition on the GA estimate by CPC.** (**A**) Scatter plot between GA and DNAm-predicted GA (CPC) across all trimesters. (**B**) Violin plot of GA acceleration (standardized residual) for each pregnancy condition.

To assess whether adverse pregnancy conditions influence the epigenetic GA estimate, we applied the CPC to placental samples associated with chromosomal abnormalities (confined placental mosaicism, diandric triploidy, trisomy 13, 16, 18 and 21), neural tube defects (anencephaly and spinal bifida), intrauterine growth restriction, maternal complications (gestational diabetes and preeclampsia), and chorioamnionitis (test, n=326). Interestingly, the CPC accurately predicted the GA of fetuses with the above-mentioned conditions (MAE=1.02, r=0.98, Figure 3A) even though the CPC was constructed using unaffected control samples only.

To test whether pregnancy conditions are associated with faster/slower epigenetic aging, we used epigenetic measures of GA acceleration that were formally defined as raw residuals resulting from regressing the DNAm GA estimate on observed GA. By definition, this residual-based measure of GA acceleration is not correlated with true GA (r=0). GA acceleration did not significantly deviate from zero for any pregnancy conditions mentioned above (Figure 3B), but we acknowledge the small sample sizes for diandric triploidy (n=3) and trisomy 16 (n=3). When restricting the analysis to placental samples from the first trimester (weeks 1 to 12), we found the CPC’s GA estimates to be slightly inaccurate, which was due to the small training set (only n=7 fetuses with GA < 12 weeks).

### Refined robust placental clock for uncomplicated term pregnancies

For researchers who are particularly interested in uncomplicated term pregnancies, we also developed a second version of the RPC using placental samples from "uncomplicated term” pregnancies (defined as GA > 36 weeks) without any known pregnancy condition.

**Figure 4:**
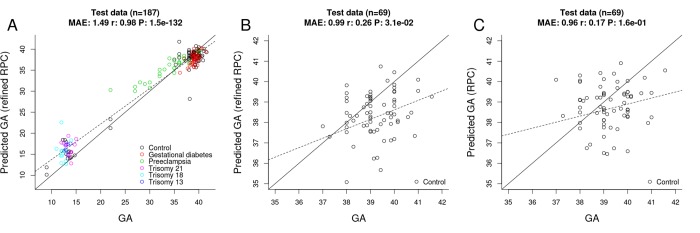
**Gestational age estimation by the refined RPC and the RPC.** (**A**) Scatter plot between observed GA and DNAm-predicted GA (by the refined RPC) – all samples from the RPC’s test data (n=187). (**B**) Scatter plot between observed GA and DNAm-predicted GA (by the refined RPC) - uncomplicated term samples from the RPC’s test data (n=69). (**C**) Scatter plot between observed GA and DNAm-predicted GA (by the RPC) - uncomplicated term samples from the RPC’s test data (n=69).

Toward this end, we selected "uncomplicated" term placental samples (n=733) from the training set used for the original RPC. Further, we restricted the penalized regression model analysis to the 558 CpGs that make up the original RPC. The penalized regression model automatically selected 395 CpG sites out of the 558 sites (Supplementary File 1). We find that the "refined" RPC for uncomplicated term pregnancies leads to highly accurate GA estimates (MAE=1.49, r=0.98, Figure 4A) in the RPC’s test set (n=187).

### Evaluating other epigenetic clocks

Using RPC’s test data (n=187), we found that previously published epigenetic clocks derived from cord blood samples or other tissues do not apply to the estimation of GA based on placental samples.

No significant correlation between GA and predicted DNAm age could be observed for clocks by Hannum (2013) [34], Horvath (2013) [23], Levine (2018) [35], and Horvath (2018) [36] (Supplementary Figure S5). However, the DNAm age estimate is close to zero for Horvath’s pan-tissue clock and the more recently developed Skin & Blood clock. Similarly, GA estimators for cord blood (Bohlin's cord blood clock [24], Knight’s cord blood clock [25]) failed to accurately predict GA in placental samples (Supplementary Figure S6). Overall, these studies demonstrate that the placenta is quite distinct from other tissues regarding the development and application of DNAm based age estimators.

### Fetal sex-classifier based on DNAm

Several GEO datasets did not report fetal sex (e.g., GSE70453, GSE73375 and GSE76641) and CpGs present on sex chromosomes. Therefore, we developed a fetal sex-classifier based RPC’s training data (n=1,102) using CpGs that are present on autosomes. Toward this end, we regressed fetal sex (binary outcome) on 441,870 autosomal CpG sites using an elastic net implemented in the glmnet R package [37]. The elastic net automatically selected 220 autosomal CpG sites. The classification accuracy was 100% for the placental test data from GSE75196 (n=24). Interestingly, the placental sex classifier turns out to be highly accurate, when applied to blood-based DNAm data from adults (e.g., an accuracy of 96% in the data from the Framingham Heart Study, n=2,356). As the sex of the fetus is typically identical to the sex of its placenta (except for rare cases of chimerism or sex-chromosome mosaicism), the sex-classifier was used to impute fetal sex in GSE66210, GSE70453, GSE73375 and GSE76641.

### Epigenome-wide association studies of gestational age

**Figure 5:**
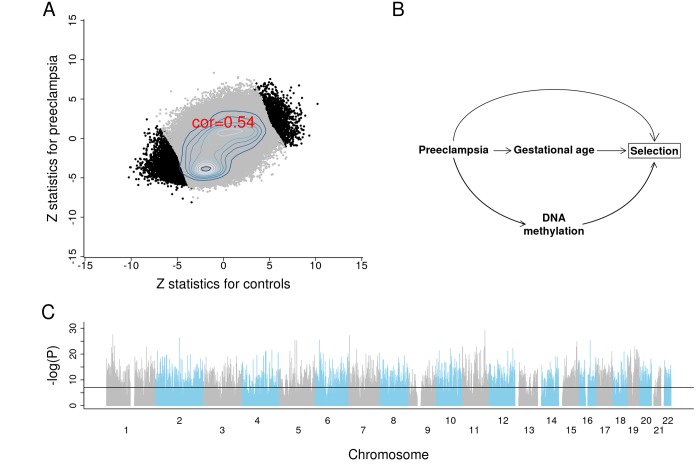
**Results of EWAS and potential confounding between DNA methylation and gestational age due to selection bias.** (**A**) Scatter plots between Z scores from controls and Z scores from preeclampsia. (**B**) The depicted minimal causal diagram under the null hypothesis of no effect of GA on DNAm. Here, the pregnancy condition (preeclampsia) would induce a spurious association between DNAm and GA, because preeclampsia could prompt earlier delivery (shorter GA) and influence DNAm. Note that the association between GA and DNAm is not due to a direct causal relationship between DNAm and GA. Rather, the association is confounded by preeclampsia. If the selection criteria differ substantially across studies, the placental clock models may not perform well. (**C**) EWAS Manhattan plot of GA.

We briefly report the results from an epigenome wide association study (EWAS) of GA to demonstrate the profound effect of GA on placental DNAm levels. To protect against confounding by preeclampsia, we conducted EWAS in two separate strata: first, for placental samples from control pregnancies (n=831); second, for placental samples from pregnancies with preeclampsia (n=70). We combined the summary statistics from the two EWAS using Stouffer's method for meta-analysis [38]. The two EWAS summary statistics presented consistent DNAm-GA correlations across 441,870 autosomal CpGs (Figure 5A).

Strikingly, 10,827 CpG sites exhibit a genome-wide significant correlation with GA (P<1E-07; Figure 5C, Supplementary File 2). Among these sites, 5,940 were in CpGs islands, 262 were in the north shelf, 1,165 in the north shore, 241 in the south shelf, and 902 in the south shore. The top four genes with the largest number of significant CpG sites were *MAD1L1* (17 CpGs), *BRD2* (13 CpGs), *INPP5A* (12 CpGs) and *RPTOR* (9 CpGs). The top 25 CpG sites and their nearest gene(s) are reported in Table 2.

**Table 2:** The top 25 CpG sites associated with GA.

CpG	Gene	Chr	Relation to UCSC CpG Island	UCSC RefGene Group	Meta Z (P) (n=901)	Z (P) of Control (n=831)	Z (P) of Preeclampsia (n=70)
cg23034799	*CADM1*	11	Island	TSS200	-11.4 (7E-30)	-10.3 (6E-23)	-4.9 (2E-06)
cg03418552	*CADM1*	11	Island	TSS200	-10.1 (6E-24)	-9.4 (8E-20)	-3.9 (2E-04)
cg21155609	*FAM167B*	1	N_Shore	1stExon	11. (3E-28)	10.2 (1E-22)	4.4 (2E-05)
cg27339550	*ZNF853*	7	Island	TSS1500	-10.9 (7E-28)	-9.2 (4E-19)	-5.9 (1E-08)
cg20025003	*TFCP2L1*	2	Island	TSS200	-10.8 (4E-27)	-9.5 (6E-20)	-5.2 (6E-07)
cg02215898		6	Island		-10.6 (3E-26)	-10.1 (2E-22)	-3.7 (3E-04)
cg11544721	*CETN3*	5	Island	Body	-10.5 (5E-26)	-10. (4E-22)	-3.7 (3E-04)
cg01152986	*SETD6;SETD6*	16	Island	TSS200	-10.5 (5E-26)	-9.7 (7E-21)	-4.2 (4E-05)
cg08757742	*RASGRF2*	5	Island	TSS200	-10.5 (6E-26)	-9.2 (6E-19)	-5.2 (6E-07)
cg26662656		15	N_Shelf		10.5 (1E-25)	8.2 (1E-15)	6.7 (2E-10)
cg13458335	*BMP8B*	1	Island	TSS1500	-10.1 (6E-24)	-9. (2E-18)	-4.6 (8E-06)
cg20630277	*MRPL23*	11	Island	Body	-10. (1E-23)	-9. (2E-18)	-4.4 (2E-05)
cg21908248	*PPP1R15B*	1	Island	1stExon	-10. (1E-23)	-9. (4E-18)	-4.5 (1E-05)
cg26940573	*ZNF566*	19	Island	1stExon;5'UTR;TSS200	-10. (1E-23)	-8.8 (9E-18)	-4.7 (5E-06)
cg13242525	*FAM86C*	11	Island	TSS1500	-10. (1E-23)	-8.2 (2E-15)	-5.9 (2E-08)
cg13512138	*CHID1*	11	Island	5'UTR	-10. (2E-23)	-8.8 (1E-17)	-4.7 (4E-06)
cg05569874	*SEMA4B*	15	Island	5'UTR;1stExon	-10. (2E-23)	-9.3 (2E-19)	-3.8 (2E-04)
cg21060796	*LAYN*	11	Island	Body	-10. (2E-23)	-8.5 (1E-16)	-5.2 (6E-07)
cg01103597	*RUNX3*	1		Body	9.9 (3E-23)	8.5 (1E-16)	5.1 (7E-07)
cg12799981	*ASCC1;C10orf104*	10	N_Shore	1stExon;5'UTR;TSS1500	-9.9 (7E-23)	-9.4 (1E-19)	-3.4 (7E-04)
cg12888127	*KNTC1;RSRC2*	12	Island	TSS1500;TSS200	-9.9 (7E-23)	-9.2 (4E-19)	-3.7 (3E-04)
cg03366925	*GLI3*	7	Island	TSS1500	-9.8 (1E-22)	-8.5 (1E-16)	-4.9 (2E-06)
cg19599862	*ZNF226*	19		1stExon;5'UTR	-9.8 (1E-22)	-8.2 (1E-15)	-5.4 (2E-07)
cg16449659	*TIGD4;ARFIP1*	4	S_Shore	TSS1500;5'UTR	-9.7 (2E-22)	-9.1 (1E-18)	-3.7 (3E-04)
cg27006129	*ZNF114*	19	N_Shore	TSS1500	-9.7 (3E-22)	-7.9 (1E-14)	-5.7 (3E-08)

The RPC had 36 epigenome-wide significant (P<1E-07) CpG sites, the CPC had 39, and the refined RPC had 32.

## DISCUSSION

Using the largest placental training set to date (n=1,102), we developed highly robust molecular estimators of GA. The robust placental epigenetic clock (RPC) is expected to perform well, even when applied to cases with adverse fetal outcomes or pregnancy complications. We developed this clock using a placenta-based training set that included several adverse conditions, including chromosomal abnormalities (trisomy and triploidy), neural tube defects (anencephaly and spinal bifida), intrauterine growth restriction, maternal complications (gestational diabetes and preeclampsia), and chorioamnionitis.

In contrast, the only other published placental clock by Mayne and colleagues was trained on a small training set (n=170). In our independent test set (n=187), Mayne’s clock under/overestimated GA according to pregnancy conditions. (Supplementary Figure S1). These systematic deviations from Mayne's clock might reflect interesting biological effects or technical artifacts (batch effects, normalization methods). Another potential limitation of Mayne’s clock is that the authors limited the eligible CpG sites to the approximately 18,437 autosomal sites on the 27K and 450K bead chips. This might explain why the Mayne’s clock uses only 62 CpG sites, whereas our RPC uses 558.

To infer biological processes under the 558 and 546 CpG sites, we conducted functional gene enrichment analyses using the Genomic Regions Enrichment of Annotation Tool (GREAT, v.3.0 [39],). However, we did not find any significant biological annotations associated with fetal aging. Elastic net regressions automatically select predictive CpG sites of gestational age (GA), but these CpG sites are not always biologically meaningful.

Our study had several limitations. First, the "observed" GA used for building these epigenetic clocks were estimated either by early pregnancy ultrasound or the LMP method. Although early pregnancy ultrasound based on fetal growth is the gold standard in a clinical setting, it is susceptible to variations in fetal size and leads to a systematic underestimation of GA in smaller fetuses [40–42].

There is also a concern that some of the training sets might be subject to systematic confounding due to adverse pregnancy conditions, as is the case for preeclampsia (Figure 5B). GA tends to be overestimated for placentas linked to preeclampsia, which is consistent with the associated pathology of advanced villous maturation, as well as previous reports of molecular signs of advanced aging [17,43]. In this hypothetical example, preeclampsia confounds the association between placental DNAm and GA (Figure 5B [44–46],). However, this type of confounding probably does not affect our placental clocks for the following reasons. First, the CPC for control samples and the refined RPC for uncomplicated term samples also accurately predicted GA even in pregnancies with known complications. Second, our EWAS of GA reveals profound associations between GA and DNA methylation levels even after stratifying the analysis by preeclampsia.

Moreover, it is possible that the RPC and the CPC might not perform well in case of non-live births was because the proportion of non-live births was extremely small amongst the third trimester samples in the training datasets, while unavoidably all first and second trimester samples are non-live births. In addition, it has been suggested that gravidity or parity may change placental physiology (e.g., higher placental weight associated with higher parity [47]) and therefore might modify the relationship between the placental epigenome and GA.

The clinical application of the RPC might be limited, because obtaining placental samples during pregnancy is highly invasive (e.g., chorionic villus sampling [48,49]). However, the existence of a predictive placental clock – the RPC – opens the possibility to develop another epigenetic clock based on cell-free fetal DNA (cffDNA). cffDNA is fragmented from placenta trophoblasts [50,51], and circulates in maternal blood during pregnancy [52]. If the development of a cffDNA clock is successful, clinicians readily estimate GA simply by collecting and analyzing maternal blood anytime during pregnancy.

## METHODS

### Study population

We collected publicly available data from Gene Expression Omnibus (GEO) using the GEOparse Python package (Python 3.6.5: Anaconda, Inc.). Table 1 details each dataset. GSE71678 examined the correlation between placental DNAm and arsenic exposures in the New Hampshire Birth Cohort Study [53]. GSE75248 examined placental DNAm in relation to newborns’ neurobehavioral outcomes [54]. GSE71719 studied the association between DNA hydroxymethylation and gene expression using placental samples [55]. The Robinson laboratory (RL) at the University of British Columbia (Vancouver, BC, Canada) transferred placental DNAm data that are publicly available in the GEO database. GSE100197 and GSE98224 were studies that aimed to find placental DNAm profiles for preeclampsia and intrauterine growth restriction in women recruited at the University of British Columbia Women’s and Children’s Hospital (Vancouver, Canada) and at Mount Sinai Hospital (Toronto, Canada), respectively [56]. GSE108567 investigated batch effects in DNAm micro array data [57]. GSE69502 explored DNAm patterns in multi-tissue samples (placental chorionic villi, kidney, spinal cord, brain, and muscle) from fetuses that were aborted due to neural tube defects [58]. GSE74738 aimed to identify differentially-methylated imprinted regions using a genome-wide approach [59]. GSE115508 compared DNAm patterns in cases of placental inflammation (acute chorioamnionitis) with those in unaffected controls [60]. GSE44667 studied the association between placental DNAm in gene enhancer regions and early-onset preeclampsia [61]. GSE49343 investigated placental DNAm with trisomy and preeclampsia [62]. GSE42409 enhanced probe annotation of Illumina HumanMethylation 450K BeadChip to facilitate biologically meaningful data interpretation [63]. GSE120250 examined the impact of assisted reproductive technology on the placental DNA methylome [64]. GSE70453 conducted epigenome-wide and transcriptome-wide analyses of gestational diabetes [65]. GSE73375 examined DNAm in the preeclamptic placenta in relation to the transforming growth factor beta pathway [66]. GSE75196 studied different DNAm patterns in patients with preeclampsia and unaffected controls [67]. GSE76641 studied the transcriptional and DNAm trajectory of 21 organs during fetal development [68].

### Measurement of DNA methylation

Either the Illumina Infinium HumanMethylation450 BeadChip or the Infinium MethylationEPIC BeadChip was used to measure DNAm level at each CpG site. The DNAm level (β-value) was the ratio of two fluorescence signals (methylated and unmethylated). The minfi R package [31] was used to preprocess all the DNAm datasets except for GS2E115508 and GSE120250 (preprocessed by Illumina’s proprietary software, Genome Studio). The preprocessing methods and probe exclusion criteria differed across studies. For example, Marsit and colleagues, the largest GEO submitter, used the funNorm, whereas Robinson and colleagues mostly used the funNorm or the SWAN (Table 1). Other GEO submitters used the BMIQ, funNorm, quanNorm, dasen, or noob. Most GEO submitters excluded probes on sex-chromosomes, near single nucleotide polymorphisms, with cross-hybridization or with a detection p-value > 0.01.

### Pre-processing of DNA methylation data

We ensured that all samples were included only one time in our training data. Some GEO datasets re-used the same samples or included technical replicates. For example, 154 samples were re-used in GSE100197, GSE108567, GSE69502, GSE74738, GSE44667, GSE49343 and RL data; and 15 technical replicates were found in GSE100197 and RL data. The sample size (N) in Table 1 refers to the counts after excluding the re-used samples and replicates.

We detected and removed outliers using the following steps: 1) we defined a gold standard DNAm profile as the inter-sample median value. For each CpG, we computed the median beta value across all placental samples. 2) The gold standard was correlated with each placental sample to calculate the Pearson correlation coefficient. 3) Placental samples were excluded if their correlation with the gold standard profile was lower than 0.9. Overall, only four putative outliers were removed from the analysis.

Missing DNAm levels were imputed with the gold standard DNAm levels. Thus, if the beta value of a CpG was missing, the missing value was imputed with the interpersonal median value across all samples. These imputations were only implemented in the training data.

### Elastic net regression of gestational age

We fit a penalized regression model using the “glmnet” R package [37]. GA was regressed on 441,870 CpG sites that are shared between the 450K and the EPIC array. The glmnet mixing parameter alpha was set to 0.5 (specifying elastic net regression), and the shrinkage parameter, lambda resulting in the minimum mean square error, was chosen using 10-fold cross-validation in the training data. The RPC automatically selected 558 CpG sites (lambda=0.0936), the CPC did 546 CpG sites (lambda=0.0892), the refined RPC did 395 CpG sites (lambda=0.0116), and the fetal sex-classifier did 220 CpGs (lambda=0.0073). The number of overlapping CpGs between the RPC and CPC was 199. Supplementary File 1 includes CpG sites and their corresponding coefficients for the RPC, CPC, refined RPC and fetal sex-classifier.

### Epigenome-wide association study of gestational age

We used the R function "standardScreeningNumericTrait" from the weighted gene co-expression network analysis R package (WGCNA [69];) to carry out a robust correlation test (based on the biweight midcorrelation) between each CpG and GA. We conducted two separate EWAS of GA: one in control placental samples (n=831) and the other in placental samples from preeclampsia cases (n=70). We computed biweight midcorrelations between DNAm levels and GA, and the corresponding Z statistics and p-values in each stratum. The Z statistics of the two sets of EWAS were combined using the weighted Stouffer’s method [38] as: $\sum Z_{i}w_{i}/\surd \sum w_{i}^{2}$, where $w_{i}$ is the square root of the sample size in the ith stratum. The corresponding p-values were computed as $2\left(1-Ö\left(\left|Z_{meta}\right|\right)\right)$. The EWAS was limited on the 411,870 autosomal probes available on both the 450K and the EPIC array platform.

### Software availability

The coefficient values of the placental clocks and the fetal sex classifier can be found in Supplementary File 1.

## Supplementary Material

Supplementary Figures

Supplementary File 1

Supplementary File 2
