# Supplementary material for: Placental epigenetic clocks: estimating gestational age using placental DNA methylation levels
Source: Aging (Albany NY). 2019 Jun 24;11(12):4238–53. doi: 10.18632/aging.102049 (PMC6628997; doi:10.18632/aging.102049)
Supplement: Supplementary Figures [file aging-11-102049-s002.pdf]

SUPPLEMENTARY FIGURES

This file includes figures that display the predictions of the RPC and other DNAm-based chronological age estimators. To elucidate the robustness of the RPC, the data points in the figures were colored according to pregnancy trait, fetal sex and ethnicity. This file includes a flow chart that describes the composition of training and test data for the CPC.

.

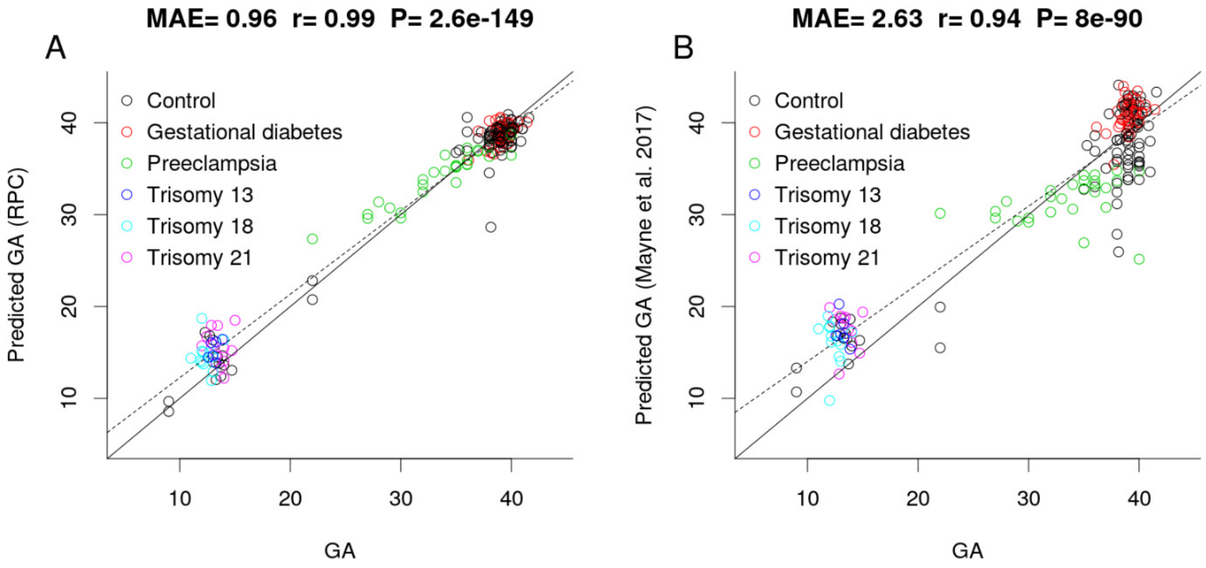

Figure S1. Gestational age estimation by the RPC and Mayne et al. (2017)'s placental clock according to pregnancy complications or congenital malformations.

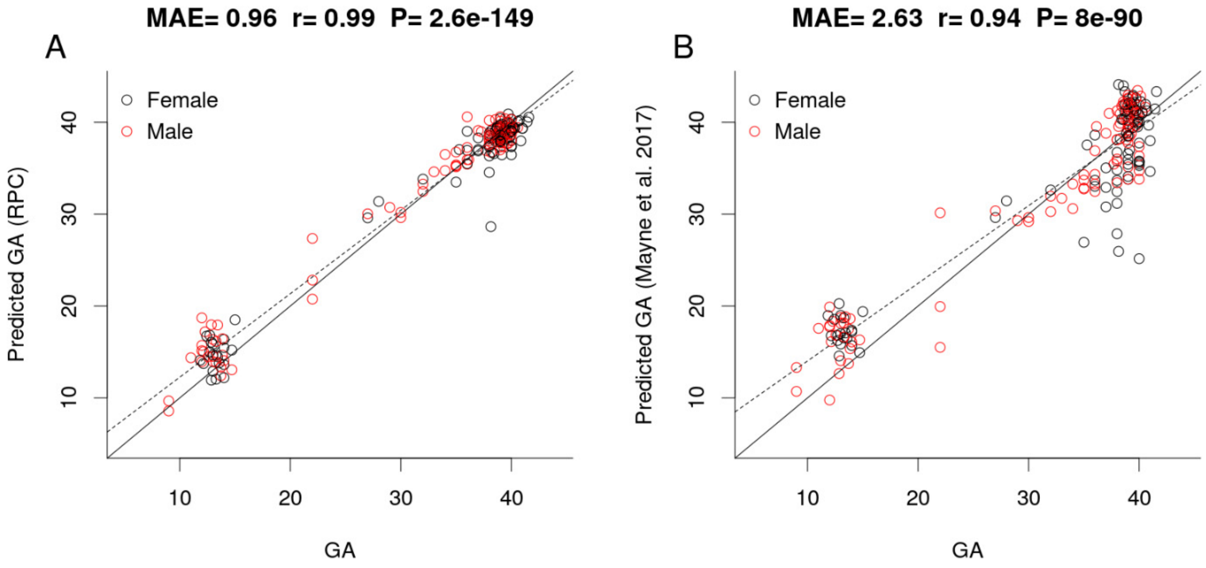

Figure S2. Gestational age estimation by the RPC and Mayne et al. (2017)'s placental clock according to fetal sex.

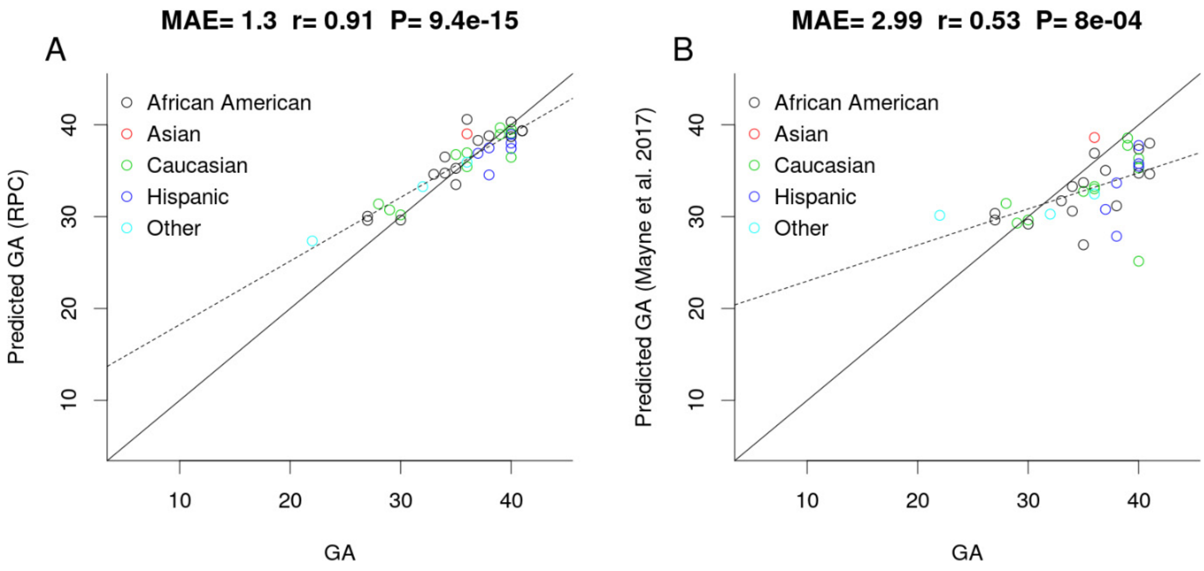

Figure S3. Gestational age estimation by the RPC and Mayne et al. (2017)'s placental clock according to ethnicity.

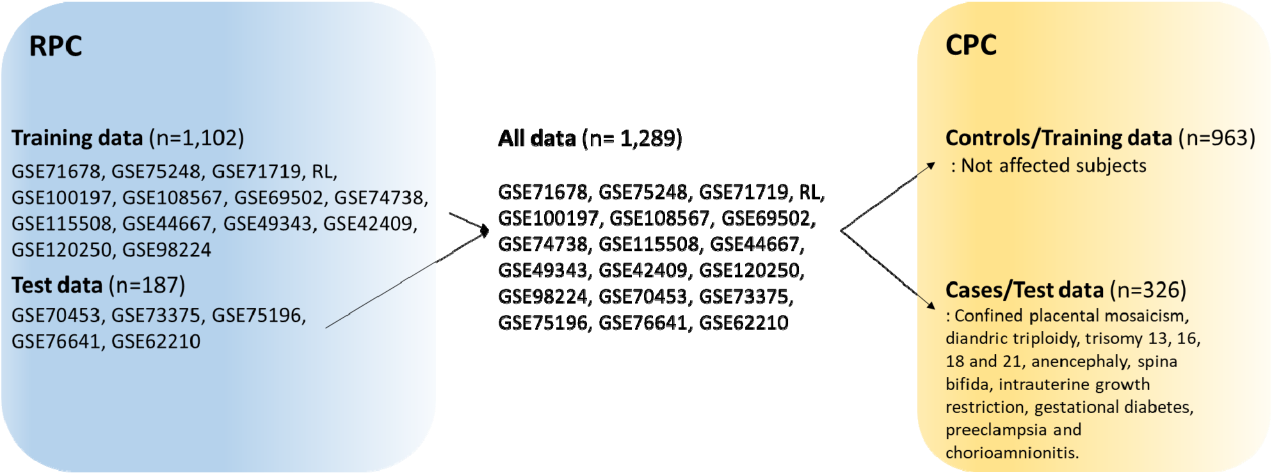

Figure S4. Forming training and test data for the CPC.

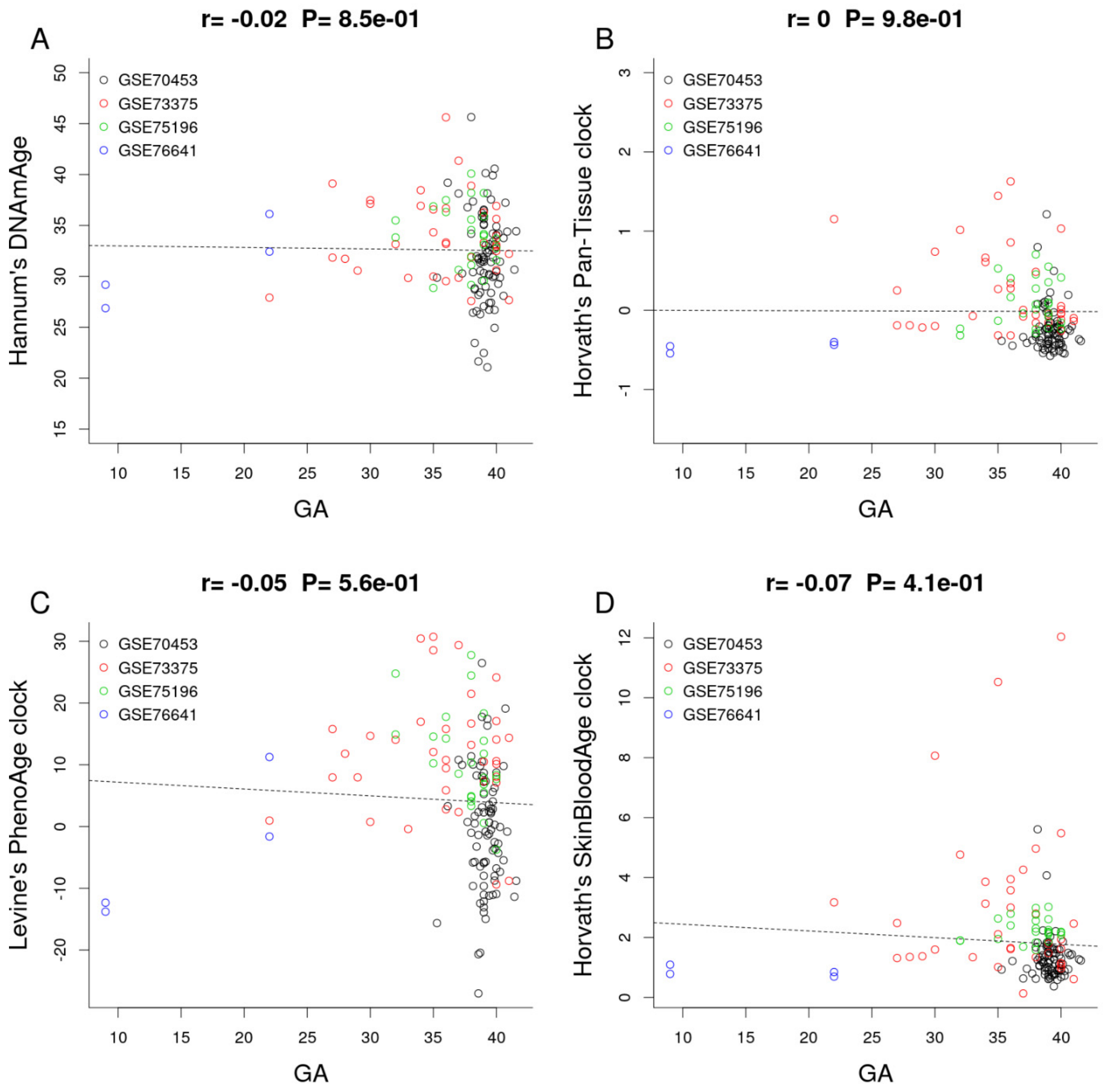

Figure S5. Gestational age estimation by different epigenetic clocks (Hannum et al. (2013)'s blood based clock, Horvath (2013)'s pan-tissue clock, Levine et al. (2018)'s PhenoAge clock and Horvath et al. (2018)'s Skin & Blood clock).

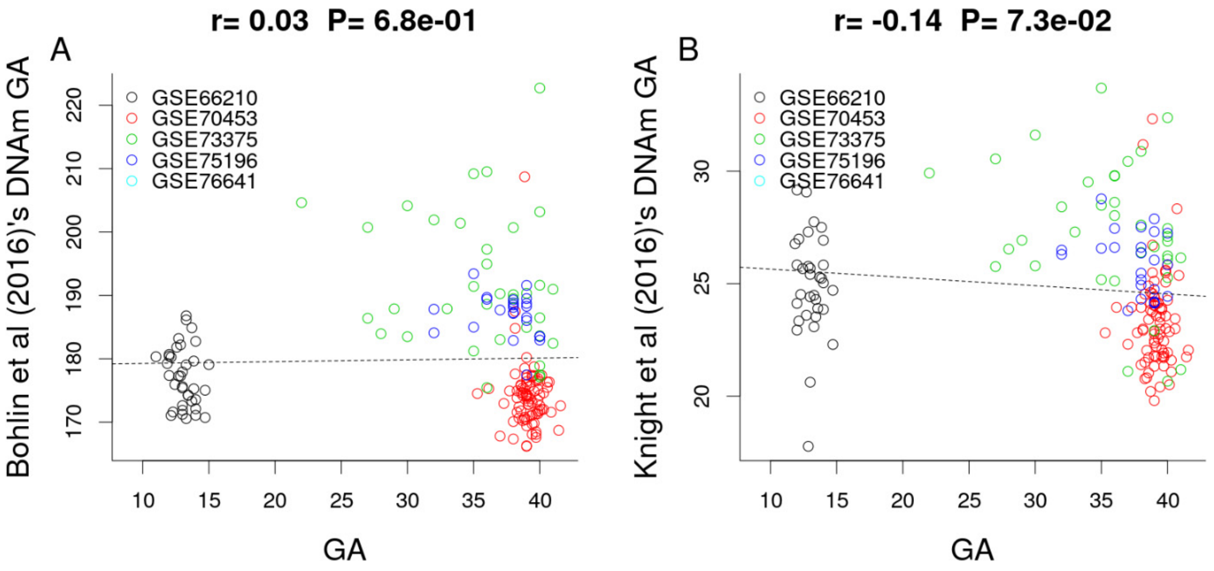

Figure S6. Gestational age estimation by the cord blood clocks developed in Bohlin et al. (2016) and Knight et al. (2016).
